# Application of the Linux cluster for exhaustive window haplotype analysis using the FBAT and Unphased programs

**DOI:** 10.1186/1471-2105-9-S6-S10

**Published:** 2008-05-28

**Authors:** Hiroyuki Mishima, Andrew C Lidral, Jun Ni

**Affiliations:** 1 Dows Institute for Dental Research, College of Dentistry, University of Iowa, Iowa City, IA 52242, USA; 2 Department of Orthodontics, College of Dentistry, University of Iowa, Iowa City, IA 52242, USA; 3 Department of Radiology, Carver College of Medicine, University of Iowa, Iowa City, IA 52242, USA; 4 Current address: Nagasaki University Global Center of Excellence Program; and Department of Human Genetics, Nagasaki University Graduate School of Biomedical Sciences, Nagasaki, Nagasaki 852-8523, Japan

## Abstract

**Background:**

Genetic association studies have been used to map disease-causing genes. A newly introduced statistical method, called exhaustive haplotype association study, analyzes genetic information consisting of different numbers and combinations of DNA sequence variations along a chromosome. Such studies involve a large number of statistical calculations and subsequently high computing power. It is possible to develop parallel algorithms and codes to perform the calculations on a high performance computing (HPC) system. However, most existing commonly-used statistic packages for genetic studies are non-parallel versions. Alternatively, one may use the cutting-edge technology of grid computing and its packages to conduct non-parallel genetic statistical packages on a centralized HPC system or distributed computing systems. In this paper, we report the utilization of a queuing scheduler built on the Grid Engine and run on a Rocks Linux cluster for our genetic statistical studies.

**Results:**

Analysis of both consecutive and combinational window haplotypes was conducted by the FBAT (Laird et al., 2000) and Unphased (Dudbridge, 2003) programs. The dataset consisted of 26 loci from 277 extended families (1484 persons). Using the Rocks Linux cluster with 22 compute-nodes, FBAT jobs performed about 14.4–15.9 times faster, while Unphased jobs performed 1.1–18.6 times faster compared to the accumulated computation duration.

**Conclusion:**

Execution of exhaustive haplotype analysis using non-parallel software packages on a Linux-based system is an effective and efficient approach in terms of cost and performance.

## Background

**Figure 1:**
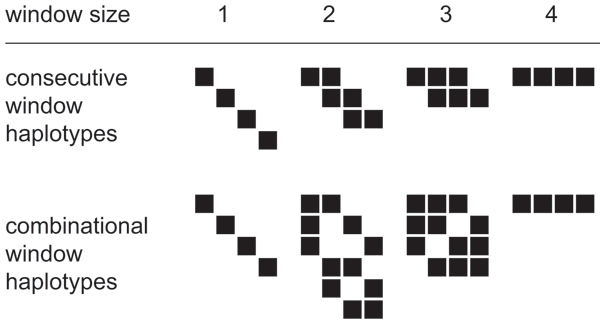
**Consecutive and combinational window haplotypes**. Examples of consecutive and combinational window haplotypes for four loci. Each column of squares indicates loci, and each row indicates haplotype windows.

Genetic association studies are a gene-discovery strategy that compares the frequency of genetic variation at a chromosomal position (locus) between cases and controls to assess whether they contribute to the disease phenotypes. Association studies are categorized into ether population-based or family-based with case-control studies being the former. The family-based association test (FBAT) is an extension of the case-control study to family trios consisting of a father, a mother and an affected child [1]. A unique feature of FBAT, which is one of the most commonly used statistical genetic packages, is that the control group is defined by the set of genetic variants that are not inherited by the offspring [2]. The recently developed Unphased program [3] extends the FBAT method using likelihood models. Conventional association studies conduct pair-wise comparisons at each individual locus. Alternatively, haplotype association analysis, which uses information from groups of multiple loci (haplotypes), can effectively narrow the disease gene location with greater power. FBAT implements haplotype analysis by its interactive command hbat, while Unphased implements them by both command-line options or graphical user interface written in Java. Haplotypes describe the linear relationship of a series of loci along the chromosome strand and are user defined by the number of loci (i.e., window size) and either consecutive or combinational windows (see figure 1). Consecutive window haplotypes (ConsWH), known as sliding window haplotypes, consist of different sets of contiguous loci at various sliding positions. Combinational window haplotypes (CombWH) consist of all possible loci combinations in a given window size. In comparison to ConsWH, CombWH can obtain greater signal-to-noise ratio by removing nuisance loci that do not distinguish cases from controls. One of disadvantage of CombWH is that larger window sizes can yield divergent results, hence failing to narrow the disease gene location. The method to analyze all ConsWH or CombWH in any window sizes are called exhaustive window haplotype analysis. This method is thought to optimally extract information by identifying disease-associated haplotypes [4].

Exhaustive window haplotype analysis requires high computational power because of the larger number of haplotypes and window sizes. Indeed, the number of haplotypes in ConsWH and CombWH analysis for the window size *n *are:

$$\begin{array}{cc} \sum_{k=1}^{n}k=\frac{n(n+1)}{2}, & \sum_{k=1}^{n}C_{n}^{k}=2^{n}-1, \end{array}$$

respectively. Unfortunately, most of commonly-used software packages for statistical genetics are not written in parallel code and rewriting them for parallel analysis requires the reliability testing of the new code. To circumvent these obstacles and still achieve higher performance, we used a queuing system to sequentially submit jobs in a parallel manner on a Linux cluster.

## Results and discussion

### Results

The FBAT and Unphased programs were utilized to analyze both ConsWH and CombWH (see table 1). To evaluate the performance, we defined fold acceleration as elapsed duration divided by accumulated duration, and acceleration linearity indexing the ratio of fold acceleration to the ideal linear acceleration as fold acceleration divided by number of compute-nodes (22, except only five were used for Unphased CombWH analysis). For ConsWH analysis using both FBAT and Unphased, fold acceleration and acceleration linearity were 13.0–18.6 and 59.1%–84.5%, respectively. On the other hand, for CombWH analysis, fold acceleration and acceleration linearity were 1.1–15.9 and 22.0%–72.3%, respectively. For Unphased ConsWH analysis, the -certain option was compared with the -uncertain option. The -certain option includes only data that is known, while the -uncertain option estimates the values of uncertain haplotypes by calculation the probability of each possibility and in doing so, requires intensive computing power.

**Table 1:** Computation Performance. Computation performance in each method. Analyzed haplotype window types were consecutive window haplotypes (ConsWH) and combinational window haplotypes (CombWH). Fold acceleration is defined as actual elapsed time divided by accumulated time for each process. Acceleration linearity is defined as fold acceleration divided by number of used compute-nodes. 22 nodes are used for analysis except five nodes for the Unphased CombWH analysis.

program	option	analyzed haplotype	window size	elapsed time	accumulated time	fold acceleration	acceleration linearity
FBAT	-e	ConsWH	1–26	2.2 min	31.7 min	14.4	65.5%
FBAT	-e	CombWH	1–5	1.9 min	30.3 min	15.9	72.3%
Unphased	-uncertain	ConsWH	1–17	69.9 day	909.0 day	13.0	59.1%
Unphased	-certain	ConsWH	1–26	13.8 min	256.1 min	18.6	84.5%
Unphased	-certain	CombWH	1–5	6.5 day	7.2 day	1.1	22.0%

**Figure 2:**
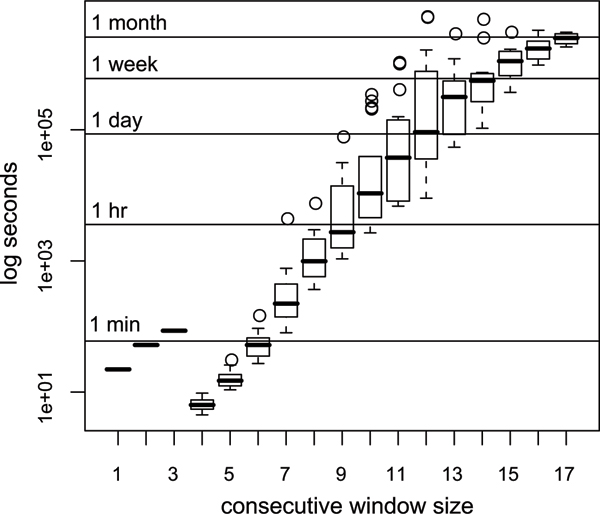
**Elapsed time for Unphased processes**. Diversity of Unphased computation duration for each window size. For window size 1–3, haplotypes sharing same window size were analyze in single processes. For window size 4–17, each haplotype was analyzed by each single process. The x-axis indicates sizes of consecutive windows. The y-axis indicates common logarithms of elapsed seconds. A boxplot indicates first quantile (bottom of a box), third quantile (top of a box), median (a bold line in a box), smallest non-outlier (a lower whisker), largest non-outlier (a upper whisker), and outliers (circles) defined as data far from more than 1.5 fold of a box size.

FBAT ConsWH analysis for window sizes consisting of 11–26 loci did not locate the disease gene position with any better precision because some haplotype frequencies were too low to be informative. Acceleration for FBAT CombWH analysis was 15.9 times higher, whereas Unphased CombWH analysis were only 1.1 times higher. This was because only five of 22 compute-nodes were used to run five Unphased processes to avoid excess overheads caused by large number of jobs that take very short time to finish. Furthermore, to observe the performance of each process using the Unphased CombWH with the -uncertain option, we compared elapsed times in different window sizes (see figure 2). Each Unphased process analyzed every haplotype. However, for window sizes of 1–3, haplotypes sharing the same window size were analyzed by single processes to avoid overheads. Analysis duration for each haplotype increased exponentially by window sizes, and the completion of some haplotypes took extremely long times.

### Discussion

Because we used programs written in non-parallel code, we adopted the process-based parallelization approach. This lower parallelization granularity is problematic when each single process takes a very long time to finish. The performance during the Unphased ConsWH analysis with the -uncertain option demonstrates the limitation of a process-based parallelization approach. Possible solutions are using higher power compute-nodes or parallelizing the code. Even though some analysis required very little time (e.g., CombWH analysis for small window size), the summation of the amount of time required to detect process termination by Grid Engine and fork a new process by the Linux kernel was not negligible when high numbers of processes were involved. Therefore, Unphased-CombWH-uncertain analyses sharing smaller window sizes 1, 2, or 3 were bundled into single processes. The Unphased-CombWH analysis of short windows, comprised by all possible combinations of 1–5 loci out of 26 total, involves > 80,000 haplotypes and results in a large number of output files. Combining analyses of all combinations for each window size made data management much more efficient, resulting in only five files, albeit with lower acceleration. Analysis with larger window sizes may require file compression, file archiving or database management softwares to optimize acceleration. Although our HPC cluster system consists of retired PCs and regular network appliances, the system was sufficient to meet our substantial statistical genetics demands. This may explained by the minimal memory required and the low network traffic between nodes by either program.

## Conclusion

The small-scale cluster developed in this study effectively accelerated the efficiency of statistical genetic analysis, saving years of time. Today, the necessity of intensive computational power is increasing at the individual and small group level. Here we show that at minimal cost, off-the-shelf hardware, open source software, and existing non-parallel statistical packages can be configured to bring HPC into the realm of small groups.

## Methods

### Cluster system

The Linux HPC cluster was build using the Rocks Cluster Distribution  version 4.3 with the SGE roll for supporting the Grid Engine job queuing system . The cluster consisted of one PC for frontend-node, and 22 PCs for compute-nodes. All the computers had Intel Pentium 4 (1.7 GHz) CPUs. The frontend-node and each compute-node was connected each other by 100BASE-T network through a switching hub.

### Dataset and statistical genetics softwares

Whole blood samples were collected from 277 extended families (546 nuclear families, 1484 persons). Subsequently, DNA was extracted from these samples, and genetic variations of single nucleotide polymorphisms (SNPs) at 26 genetic loci were characterized for each individual. The results obtained 172k-byte pedigree data file for following analysis. For the FBAT program, the original Linux executable was installed on the cluster. The hbat -e interactive command of FBAT was used for ConsWH and CombWH analysis, and the -e option was implemented to account for the bias introduced by studying multiple members from the same family. For the Unphased program, its version 3.0.10 source code was recompiled with the GNU C compiler with the option -march = pentium4 -O2 for the CPU-specific optimization. Unphased was used with -uncertain and -certain options from the command-line instead of its graphical user interface written in Java. The former option includes ambiguous genetic data to increase the sensitivity, whereas the later option evaluates only known genetic data, resulting in much quicker analysis.

### Array job submission and execution

**Figure 3:**
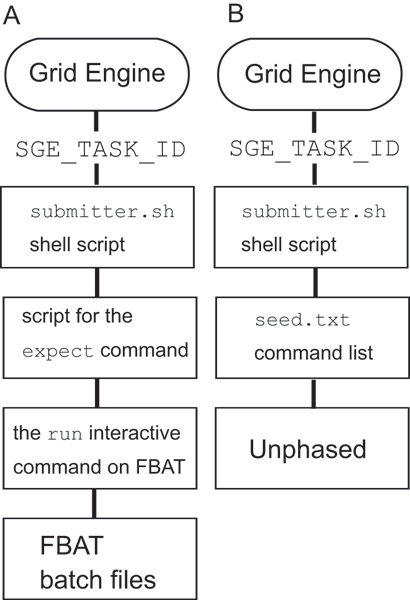
**Array job execution scheme**. Scheme of array job execution using the Grid Engine for FBAT (A) and Unphased (B). The Grid Engine assigns array job ID to an environmental variable SGE_TASK_ID. Then, script execution is cascaded.

Haplotype analysis was separated into multiple processes and submitted into a Grid Engine queue as array jobs to distribute each compute-node. Figure 3 shows the sequence of array job submission and execution is the followings. Array jobs were submitted to the Grid Engine job queue by the command qsub -t *start*: *end *submitter.sh, whereupon the Grid Engine assigned sequential array job IDs to the SGE_TASK_ID environment variable using the range defined by *start *and *end*. Subsequently, Grid Engine invoked the shell script submitter.sh to initiate the FBAT and Unphased statistical packages. For FBAT, submitter.sh invoked the expect command, which automatically provides commands according to the expect script in interactive manner, a requirement of FBAT. The expect script invoked FBAT and inputs the run interactive command to read a FBAT batch file. The FBAT batch file containing hbat interactive commands indicating the subject haplotypes were prepared by a Ruby script. To avoid memory allocation problems, we restricted the number of hbat commands for a FBAT process to 500. For Unphased, submitter.sh read a line indicated by SGE_TASK_ID from a text file seed.txt. The seed.txt file prepared by a Ruby script contained command lines for Unphased indicating haplotypes to analyze. FBAT and Unphased output their results into the Unix standard output. Grid Engine redirected them into the directory indicated in submitter.sh with the filename applying the rule of "results.o*job_ID.array_job_ID *".

### Optimization of analysis parameters

The window sizes for CombWH analysis were limited to 1–5 loci because the results of larger window sizes were divergent. The maximum number limit of array jobs (max_aj_jobs) was changed from default 75000 to zero by the qconf -mconf command. To increase the efficiency of job distribution, the flush time of Grid Engine (reporting_params/flush_time) was also optimally decreased by the the qconf -mconf command from default 15 sec to 5 sec.

## Competing interests

The authors declare that they have no competing interests.

## Authors' contributions

HM conceived the study, built the Linux HPC cluster, and wrote scripts for data acquisition and computation. ACL designed the genetic study, coordinated collection of the human biological samples and conducted genetic analysis. JN built the Linux HPC cluster, and conducted computational performance evaluation. All authors contributed to writing the manuscript.
